# The Utility of Platelet and Coagulation Testing of Antithrombotics: Fusing Science with Patient Care

**DOI:** 10.1002/ddr.21119

**Published:** 2013-11-15

**Authors:** Lisa K Jennings, Jayaprakash Kotha

**Affiliations:** The University of Tennessee Health Science Center and CirQuest LabsMemphis, TN, USA

**Keywords:** platelets, thrombosis, bleeding, platelet testing, coagulation testing, antithrombotics

## Abstract

**Table:** 

Strategy, Management and Health Policy				
Enabling Technology, Genomics, Proteomics	Preclinical Research	Preclinical Development Toxicology, Formulation Drug Delivery, Pharmacokinetics	Clinical Development Phases I-III Regulatory, Quality, Manufacturing	Postmarketing Phase IV

There is an increasing need for the standardization of platelet function and coagulation testing for the assessment of antithrombotic therapies. Investigators continue to strive to identify ideal laboratory testing and monitoring procedures for acquired and inherited platelet function defects as well as for evaluating patient status when treated with existing or emerging antithrombotics. These therapies are used primarily in the treatment of ischemic complications. In patients receiving antithrombotic therapy, the balance between hemostasis and thrombosis is a challenge as there is an ongoing risk for bleeding when patients are receiving antiplatelet agents or anticoagulants to lessen their risk for secondary thrombotic events. There are several diverse tests for monitoring anticoagulant therapy; however, as new agents are developed, more specific tests will be required to directly assess these agents in relationship to overall coagulation status. Research in the platelet biology field is ongoing to provide point-of-care methodologies for the assessment of platelet reactivity in terms of both bleeding and thrombosis risk. Currently there are no instruments that reliably assess the risk of bleeding. The challenges that routinely faced are the complexity of physiology, the need for standardization of platelet testing methodology, and the necessity for appropriate interpretation of the test results.

## PLATELETS: FUNCTION OVERVIEW

Platelets are anucleate blood cells that have a critical role in hemostasis and thrombosis. They are derived from the bone marrow myeloid precursor cell, the megakaryocyte, and are generated by a demarcation and fragmentation of the megakaryocyte protoplasm [Italiano and Hartwig, 2006]. Once released into circulation, their life span is approximately 7–10 days. Measuring only 2–4 μm in diameter, platelets contain many storage granules, a continuous membrane structure, diverse cell surface receptors, and signaling molecules that direct platelet adhesion, activation, and aggregation as well as coagulation [White, 2006]. The procoagulant phospholipid surface of activated platelets is responsible for the generation and propagation of thrombin, the final step in the coagulation cascade that is responsible for the conversion of fibrinogen to fibrin. Fibrin is subsequently polymerized to form a mesh-work that contributes to the generation of the hemostatic plug formation to arrest bleeding [Jennings, 2009a]. In addition, thrombin is a highly potent agonist of platelets, and its cleavage of PAR1 and PAR4 receptors can lead to irreversible platelet aggregatory activity [Jennings, 2009b]. Other key platelet agonists are fibrillar collagen, adenosine diphosphate (ADP), and thromboxane A_2_. Platelets are also responsible for clot retraction that leads to consolidation of the wound area and promotes healing [Kasahara et al., 2013]. Platelet function testing is performed in the routine evaluation of bleeding disorders and monitoring of antiplatelet therapies. Platelet function evaluation is becoming more prevalent as platelet surface antigens or their granule constituents are also associated with inflammation, vascular remodeling, tumor growth and distal metastasis, and host defense mechanisms [Harrison and Lordkipanidze, 2013].

## PLATELET AND COAGULATION ABNORMALITIES

Platelet function status can profoundly affect patient well-being. Dysfunctional platelets contribute significantly to bleeding diatheses found in several inherited and acquired syndromes, including MYH9-related disorders [Lages and Weiss, 1988; Flick et al., 1991; Rao, 1998; Pallotta et al., 2005; Lhermusier et al., 2011]. On the other hand, highly reactive platelets contribute to complications in myeloproliferative disorders, polycythemia vera, acute phase reaction as well as in coronary artery disease, peripheral arterial disease, and stroke [Ten Cate, 2011]. Many efforts in academia and industry have been focused on the development of agents that inhibit either platelet function or coagulation to reduce the risk of ischemic complications. These agents have been widely used in the cardiovascular arena [Saucedo and Jennings, 2008; Jennings, 2009b]. Interestingly, little advancement has been made in the development of agents that are specifically targeted to the arrest of bleeding. Although several anticoagulants and antiplatelet agents were discovered and evaluated initially by in vitro and ex vivo testing, specific laboratory methods for assessing thrombotic or bleeding risk have either not been developed or adequately standardized to be widely adopted in the clinical setting. Thus, the role that platelet function testing plays in personalized medicine is still under debate [Petricevic et al., 2013]. For anticoagulants, basic prothrombin (PT) or activated partial thromboplastin time (aPTT) testing has served as an initial screening of anticoagulants [Bauer, 2010]. For newer agents, e.g., Factor Xa inhibitors, anticoagulant effects are typically evaluated using specific anti-Xa assays that more directly assess the drug effect on coagulation [Favaloro et al., 2011; Tripodi, 2013]. As other targets are identified for the prevention of thrombosis like inhibitors for Factor IXa or Factor XIIa, specific tests may be necessary to better assess effects of these agents on overall anticoagulant status.

Thrombin has a central position in the blood coagulation pathway and serves many functions in the vasculature, including generation of Factor Xa, fibrin formation, interaction with thrombomodulin, and cleavage of the protease-activated receptors on platelets [Ten Cate, 2011]. Studies show an association between thrombin generation in plasma and atherosclerotic disease [Bernhard et al., 2010]. Patients with acute myocardial infarction (MI) have increased thrombin generation for several months post their ischemic event [Merlini et al., 1994; Orbe et al., 2008; Undas et al., 2009; Smid et al., 2011]. Interestingly, although the relevance of increased thrombin generation in the acute phase of MI is uncertain [Ten Cate, 2011], data from the Atlas ACS2 TIMI 51 study showed that in patients with recent acute coronary syndrome (ACS), treatment with the Factor Xa inhibitor, rivaroxaban, reduced the risk of the composite endpoint of death from cardiovascular causes, MI, or stroke compared with control [Mega et al., 2012]. Although this beneficial effect was associated with increased bleeding rates, the addition of very low doses of anticoagulatant to harness thrombin generation may represent a new paradigm in treatment of ACS. Continued investigation of thrombin generation and the role it plays in arterial vascular diseases is warranted.

In regard to hypercoagulability, venous thromboembolism (VTE) affects about 0.1% of the US population and may result in more than 50 000 deaths annually [Khor and Van Cott, 2009]. Among the patients with an unprovoked VTE, a large percentage will have an acquired or a hereditary risk factor. Determination if the patient fulfills the criteria for the antiphospholipid antibody syndrome is the first and foremost evaluation to carry out [Bauer, 2010]. Other hypercoagulable states that predispose patients to venous and, in some cases, arterial thrombosis include activated protein C resistance/Factor V Leiden, prothrombin G20210A mutation, deficiencies of protein C, protein S, or antithrombin [Khor and Van Cott, 2009]. In addition, thrombosis can be due to acquired determinants such as cancer, long term in-dwelling catheters, and as a risk of pregnancy [Schaafer et al., 2003]. Laboratory testing using either functional or antigenic assays or DNA detection methods do have a key role in identifying an increasing number of hypercoagulable conditions. Recommendations for laboratory evaluation of hypercoagulability have been thoroughly outlined by Khor and Van Cott [2009]. For example, the d-dimer test is a valuable laboratory test for the evaluation and management of a variety of thrombosis-related disorders, such as VTE and disseminated intravascular coagulation. Although the d-dimer test has been used primarily to exclude VTE in symptomatic patients, elevated d-dimer levels have been recognized as a determinant of recurrent risk following a first unprovoked episode of VTE [Tripodi, 2011]. A meta-analysis of seven studies that included almost 1900 patients who completed ≥3 months of anticoagulation reported that a d-dimer level of <500 ng/ml was associated with a 3.5% annual risk of VTE recurrence, whereas a level >500 ng/ml was associated with an 8.9% risk in each of the first 2 years [Verhovsek et al., 2008]. Challenges with these types of analyses are the lack of agreement and the variability in clinically available d-dimer assays that must be reconciled by a d-dimer standard or the utilization of a central laboratory that utilizes a quantitative method and establishes in-house cutoffs for a positive d-dimer test.

## EVALUATION AND MANAGEMENT OF ANTICOAGULATION

Even though there are published recommendations for many anticoagulation management problems, there continues to be a need for randomized trials or further studies to establish best practices for use of both oral and parenteral anticoagulants and patient education. Although new and emerging anticoagulants may not require routine laboratory monitoring, such testing may be required on an as needed basis, particularly for the elderly and renal impaired patients, as anticoagulant effects cannot be assessed solely based on drug dosage. It is a misconception to think that there will not be a laboratory role in the management of treated patients. For example, patients admitted to emergency departments with either thrombosis or adverse bleeding should be tested for coagulation status. Typically for the newer direct oral anticoagulants, rivaroxaban and apixaban, the anti-Xa test is the most promising; for the thrombin inhibitor, dabigatran, the dilute thrombin time (TT) or ecarin clotting time, is preferred [Tripodi, 2013].

## EVALUATION OF PLATELET ABNORMALITIES

Platelet function testing options are as diverse as the coagulation tests currently being utilized primarily because several factors contribute to global platelet reactivity profile. For example, upon plaque rupture, platelets are recruited to a vulnerable site and adhere to exposed subendothelial collagen to initiate platelet adhesion and activation. Upon subsequent release of platelet storage granules, the contents not only aid in stabilizing adherent platelets but also serve to recruit nearby platelets to form a platelet thrombus. Thrombin generation due to the activation of the coagulation cascade converts fibrinogen to fibrin that binds to and stabilizes the aggregate or thrombus. Because these events are localized to one specific area of a blood vessel, evaluation of thrombosis status is challenging to perform by ex vivo blood testing.

In regard to platelet abnormalities, there are many methods that address complexities of platelet function. Patients presenting with bleeding diatheses are primarily investigated for platelet storage pool defects or defects in the release of storage granules as these defects can result in mild to moderate bleeding phenotypes. Lumiaggregometry is the accepted gold standard for assessment of platelet function abnormalities associated with bleeding risk [White et al., 1992; Lowe, et al., 2013]. In addition to storage pool disorders or platelet release defects, there are other conditions or inheritable platelet disorders that can be attributed to platelet function deficiencies such as Glanzmann's thrombasthenia, afibrinogenemia, Bernard–Soulier syndrome, or von Willebrand's disease [Nurden and Nurden, 2011].

Currently, there are no platelet function tests that can demonstrate bleeding risk when patients are administered antiplatelet therapies such as aspirin, or P2Y_12_ or GPIIb-IIIa antagonists. Platelet function testing clearly demonstrates the effect of these drugs on platelet reactivity and has been critical in demonstrating the effectiveness of a drug in blocking its target. Furthermore, platelet function studies, especially light transmission aggregometry (LTA), have historically been very informative for the in vitro evaluation of drug pharmacodynamics such as dose response, variability of response, and extent of inhibition. Factors that affect platelet aggregation testing results include drugs such as aspirin and anti-inflammatory drugs, clopidogrel and other P2Y_12_ antagonists (prasugrel and ticagrelor), and imidazole. There are also many other drugs that are not specifically targeted to platelet inhibition but can affect platelet function testing such as antibiotics, decongestants, and antidepressants. In addition, platelet count, plasma pH, blood sample temperature, fibrinogen concentration, and the anticoagulant used during venipuncture influence testing parameters and results. Phlebotomy technique, mixing of the blood with anticoagulant, sample transit to the laboratory, and laboratory handling and processing can introduce variability in platelet function testing [White and Jennings, 1999].

Platelet function testing presents a logistical challenge to a testing facility or clinical site as these tests require on-site testing in contrast to other assessments for platelet reactivity such as cell surface antigen expression, phosphorylation of vasodilator-stimulated phosphoprotein (VASP), or soluble biomarkers. Even the latter tests may require manipulation or processing beyond that typically carried out in the clinical setting. As discussed above, most testing of platelet function has been traditionally performed to assess a bleeding diathesis; however, the development of antiplatelet agents and the compelling need to better understanding and identify risk factors for thrombosis have resulted in increased research and development in this area of platelet function. The challenges that we routinely face are the complexity of platelet physiology, the need for standardization of platelet testing methodology, and the necessity for appropriate interpretation of the test results. For the above reasons, a careful consideration of platelet function testing must be carried out, especially in cases of diagnosis and management of patients.

## PLATELET FUNCTION TESTS AND THEIR UTILITY

Most platelet function tests are focused on specific aspects of platelet function and are not considered global tests of overall platelet reactivity.

LTA is primarily regarded as the gold standard for platelet function testing. Invented in the 1960s, LTA measures the formation of platelet aggregates [Born, 4]. As a suspension of platelets in plasma (platelet-rich plasma [PRP]) is typically turbid, the formation of aggregates due to activation by an agonist added exogenously to the PRP results in increased light transmission through the platelet suspension. The extent of light transmission corresponds to the extent of platelet aggregation. The utility of this assay is that multiple parameters, in addition to measuring the extent of platelet aggregation, such as baseline reactivity, onset to measureable response, shape change, rate of aggregation, and aggregate stability, may also be assessed within a single test [Jennings and White, 2006]. Some aggregometers are also capable of simultaneously assessing the release or secretion of adenine nucleotides from platelet dense granules [White et al., 1992]. There have been reports of the utility of LTA in evaluating bleeding and thrombotic risk [Harrison and Lordkipanidze, 2013]. This method has been the mainstay for evaluation of antiplatelet therapies for the treatment of acute and chronic coronary artery disease. Several publications provide assistance in standardization of LTA assessments [White and Jennings, 1999; Hayward, 2008; Mezzano et al., 2009; Harrison and Lordkipanidze, 2013].

Whole blood aggregometry (WBA; Chronolog, Haverton, PA, USA) is attractive as it avoids processing blood for PRP [Cardinal and Flower, 1980]. In principle, WBA is a different test from conventional LTA in that the test measures a change in resistance between two adjacent electrodes as platelet adhere and aggregate in response to an exogenous agonist. In addition to the Chronolog WBA instrument, the Multiplate (Roche Diagnostics, West Sussex, UK) has been adopted in some clinical laboratories [Seyfert et al., 2007]. The results of the WBA testing are different between these two instruments and do not correlate with LTA. Although there is an increase interest in the use of WBA, these instruments are not recommended currently for the identification of congenital or acquired platelet disorders or for monitoring effects of antithrombotic therapies.

The VerifyNow (Accumetrics, San Diego, CA, USA) point-of-care (POC) test has been used in clinical evaluation or monitoring of GPIIb-IIIa or P2Y_12_ antagonists, particularly in the setting of periprocedural percutaneous coronary intervention (PCI) [Van Werkum et al., 2008]. The test is based on the ability of a drug to inhibit the agglutination and aggregation of fibrinogen-coated beads by activated platelets. The response unit test result may be favored over the calculated percent inhibition test result, though neither are substantially equivalent to LTA, especially at lower drug levels [White et al., 2004; Breet et al., 1962]. Although this POC test has been useful in demonstrating high levels of platelet inhibition, studies using the VerifyNow as a means for dose adjustment of P2Y_12_ antagonists to reduce secondary events in CAD were not proven to be of added value [Price et al., 2011; Trenk et al., 2012]. This instrument is specifically designed to evaluate three antiplatelet drugs (aspirin, clopidogrel, or GPIIb-IIIa antagonists) individually.

The Platelet Function Analyzer (PFA-X00, Siemens Diagnostics, NY, USA) device is a whole blood (WB) cartridge-based assay in which anticoagulated blood is aspirated through an aperture that is coated with specific platelet agonists [Favaloro, 2002]. The coating contains either collagen and ADP or collagen and epinephrine. Using high shear conditions to mimic an occlusive vessel, the machine measures the drop in blood flow rate in response to agonist exposure and the time necessary to fully occlude the aperture. Due to the assay design, the PFA has been primarily used as a companion diagnostic tool for the diagnosis of type II and type II von Willebrand disease (vWD) [Favaloro, 2002]. The PFA device may have limited utility in the diagnosis of increased thrombotic risk or mild platelet function defects [Harrison et al., 2011].

Flow cytometry of platelets has become a critical research and clinical tool in the study of platelet biology and the analysis of platelet function in many aspects of vascular biology [Michaelson et al., 2006]. Examples of the utility of flow cytometry for platelet studies include platelet antigen surface expression (e.g. GPIb, GPIIb-IIIa) and the dynamic expression of markers for platelet activation (p-selectin), activation of GPIIb-IIIa using PAC-1 and its occupancy by either fibrinogen or drug antagonists using D3, the assessment of platelet–leukocyte aggregates, calcium influx or mobilization, secretion, microparticle formation, or receptor-mediated signaling such as the phosphorylation status of VASP [Wall et al., 1995; Jennings et al., 1989; Jennings and White, 1998; Chandler et al., 2009; Deibele et al., 2010]. The utility of this approach is limitless; however, the techniques, assessments, and interpretation of results require individuals who are experts in the field of platelet biology. As methods for sample fixation are improved, this method will gain wider application in the clinical setting when LTA testing alone is inadequate to assess overall platelet function.

Thromboelastography (TEG; ROTEM, TEM Innovations, Munich, Germany) is used to assess clot formation, including the kinetics of clotting, clot strength, and lysis that involves globally platelet function and coagulation [Scharbert et al., 2009; Stafford and Weitzel, 2013]. This test has been traditionally used in the surgical setting as a screen for bleeding risk. However, a task force of investigators (TRG/ROTEM Working Group) suggests that there is significant interlaboratory variation. Both the TEG/ROTEM and the Platelet Mapping System are not currently recommended for platelet function testing in the clinical setting.

There are other tests that have been employed for platelet function testing and investigated in terms of their utility in assessment of either bleeding or thrombosis risk. These tests include the Cone and Plate Analyzer (Matis Medical Inc., Beersel, Belgium), 96-well microtiter plate assays for platelet aggregation testing, platelet counting using the Plateletworks (Helena Laboratories, Beaumont, TX) assay and the Ichor Hematology Analyzer (Helena Laboratories), bench assays for platelet secretion and thromboxane B_2_ generation, and soluble biomarkers that measure the release or shedding of platelet-derived proteins [Ikeda et al., 1995; White et al., 2004; Fong et al., 2011]. Biomarker studies may prove informative and shed light on possible relevant targets for either diagnosis or treatment of platelet-related disorders. Clinical trials that include biomarker studies may assist in the identification of new drug targets or serve as surrogates for drug effect. Continued research and clinical experience in this area will be necessary in order to determine the specificity and sensitivity of this approach.

## THE FUSION OF SCIENCE WITH PATIENT CARE

In spite of the above limitations surrounding platelet function testing, there is evidence to support that assessment of platelet reactivity may correlate with risk of thrombosis in ACS [Aradi et al., 1]. Several studies have linked high platelet reactivity to increased risk of thrombotic complications, and data from randomized trials show that for patients deemed at a higher risk for thrombotic events, an intensified platelet inhibition strategy can considerably reduce the risk of ischemic events without increasing bleeding complications [Trenk et al., 2012; Brilakis et al., 2013]. These data suggest that high platelet reactivity alone is indeed a marker for higher risk for thrombosis and that this risk may be adjusted in ACS patients undergoing PCI. We continue to understand and explore better ways of assessing platelet function as it pertains to clinical bleeding and thrombosis. Although there is continued development of more global assays for platelet reactivity monitoring including POC instrumentation, we are also venturing into the realm of personalized medicine that will take advantage of both proteome and genome arrays [Watson et al., 2013]. An early example of these efforts includes the focus of the cytochrome P450 2C19 allele and its role in clopidogrel metabolism. There continues to be controversy how genetic variations in this allele contribute to either clopidogrel efficacy or associated risks of bleeding [Grove et al., 2013]. Regardless of the outcomes of more specialized testing, the fact remains that the goal is to devise testing that will correlate to the clinical outcome of patients both on the efficacy and safety sides of patient care.

In summary, there is a need for a broader utilization of specific tests and biomarkers to assess platelet and endothelial cell functions as well as coagulation and fibrinolysis for assessment of the clinical complications of bleeding as well as for prothrombotic states or thrombosis. Strategic implementation of testing and increasing collaborations between industry, private sector value-added research organizations, and academic scientists are needed to improve our understanding and assessment of hemostasis and thrombosis that spans preclinical drug development to routine patient care.
